# Integration of multi-omics technologies for crop improvement: Status and prospects

**DOI:** 10.3389/fbinf.2022.1027457

**Published:** 2022-10-19

**Authors:** Ru Zhang, Cuiping Zhang, Chengyu Yu, Jungang Dong, Jihong Hu

**Affiliations:** State Key Laboratory of Crop Stress Biology for Arid Areas, College of Agronomy, Northwest A&F University, Yangling, Shaanxi, China

**Keywords:** multi-omics, crop improvement, integration, artificial intelligence, precision breeding

## Abstract

With the rapid development of next-generation sequencing (NGS), multi-omics techniques have been emerging as effective approaches for crop improvement. Here, we focus mainly on addressing the current status and future perspectives toward omics-related technologies and bioinformatic resources with potential applications in crop breeding. Using a large amount of omics-level data from the functional genome, transcriptome, proteome, epigenome, metabolome, and microbiome, clarifying the interaction between gene and phenotype formation will become possible. The integration of multi-omics datasets with pan-omics platforms and systems biology could predict the complex traits of crops and elucidate the regulatory networks for genetic improvement. Different scales of trait predictions and decision-making models will facilitate crop breeding more intelligent. Potential challenges that integrate the multi-omics data with studies of gene function and their network to efficiently select desirable agronomic traits are discussed by proposing some cutting-edge breeding strategies for crop improvement. Multi-omics-integrated approaches together with other artificial intelligence techniques will contribute to broadening and deepening our knowledge of crop precision breeding, resulting in speeding up the breeding process.

## Introduction

Food security is the major issue for humans in the world nowadays. The three major grain crops, rice, wheat, and maize, have fed billions of people. However, the climate change, lack of arable land, and population expansion have led to food shortages, which require constant improvement in technologies of plant breeding (Huang et al., 2022). To date, crop breeding and improvement have achieved three major stages: phenotype-based artificial selection, hybrid breeding, and molecular breeding [marker-assisted selection (MAS) and genetically modified (GM)] (Shen et al., 2022). Thus, to feed the increasing population, new technologies, such as multi-omics, artificial intelligence (AI), and genome editing, are gradually widely used for plant breeding (breeding 4.0) to precise design. The goal of crop improvement is to select favorable alleles with high yield, good quality, and tolerance to biotic and abiotic stresses to promote the breeding of elite varieties. Using the omics technologies, new breeding strategies were further developed, such as genomic selection (GS) technology which is based on genomic estimated breeding value (GEBV) (Crossa et al., 2017).

Next-generation sequencing (NGS) technologies, including genomics, resequencing, functional genomics, transcriptomics, metabolomics, and epigenomics, have been widely applied in crop improvement. Clearly, the single-omics approach suffers from limitations that affect its sensitivity or specificity. Integration of multiple-omics technologies can overcome some of these limitations. With the acquisition of abundant sequencing data, the integrative analysis of multi-omics has become a usual method to study the genes that control important agronomic traits in crops. The association analysis combined with multi-omics makes full use of the data of comprehensive analysis and the verification of the selected core data with application in breeding. Multi-omics approaches with cutting-edge technologies such as precise genome editing tools will not only identify functional genes in a large scale to reveal the molecular mechanism of plant development and response to stress, but also provide new strategies for crop improvement. Here, we summarize current progress in this field made by multiplex omics technologies and provide a perspective for the future.

## Integration of genomics and phenomics

During the past decades, sequencing technologies have been greatly reformed and developed. Thus, high-quality reference genome sequences of many crops have been generated and improved. Based on these reference genomes, resequencing of lots of crop accessions can obtain millions of genetic variations and identify functional genes for agronomic traits during the crop domestication and improvement, such as rice, soybean, maize, cotton, and so on (Xu et al., 2012; Zhou et al., 2015; Wang et al., 2017; Wang et al., 2020).

In recent years, dozens of powerful tools for quantitative trait loci (QTL) mapping have been developed based on deep sequencings, such as restriction-site associated DNA sequencing (RAD-seq), genotyping-by-sequencing (GBS), bulked segregant analysis sequencing (BSA-seq), and specific locus amplified fragment sequencing (SLAF-seq) (Bundo et al., 2022). These mapping approaches used the genetic map or limited number of SNPs to seek the candidate genomic regions and genes for the traits. The disadvantage of these strategies is that the candidate region is large and mainly dependent on the parental traits as well as not precise enough.

Combining the genetic variations (genotype) with phenotype, a genome-wide association study (GWAS) could dissect complex traits and identify candidate genes with natural variations based on millions of SNPs (Fang et al., 2017a; Fang et al., 2017b). In rapeseed, a total of 628 associated loci were detected for 56 agronomically important traits in 403 diverse accessions, including the *BnRRF* gene for 1000-seed weight (Hu et al., 2022). These genetic loci and causative candidate genes provide a valuable genomic resource for important traits in crops, which will facilitate crop improvement and variety development. Furthermore, pan-genome could cover much more genetic variations, and graph-based pan-genome can provide abundant genetic resources for plant breeding (Shen et al., 2022).

The genotyping platforms combined with high-throughput phenotyping could achieve valuable genetic information for complex traits in crops with standardization and high reproducibility (Zhang et al., 2017). For plant phenomics, advanced sensors, machine vision, and automation technology have been used for phenotyping, including unmanned aerial vehicles (UAV), hyperspectral imaging, and computed tomography (CT) (Yang et al., 2020). Using the automatic phenotyping platform, high-throughput phenotyping data were obtained in several crops such as rice, maize, and rapeseed (Yang et al., 2014; Guo et al., 2018; Li et al., 2020; Wu et al., 2021). For instance, using a high-throughput multiple optical phenotyping system, image-based traits (i-traits) were extracted and detected 2,318 candidate genes by GWAS for drought response in maize (Wu et al., 2021). Also, based on the time-resolved i-traits in rapeseed, the genetic architecture of plant growth and yield were dissected (Li et al., 2020). Using CT, tillering in rice was modeled for approximately 700 associated traits (Wu et al., 2019). High-throughput phenotyping with deep learning analysis pipelines, such as deep plant phenomics (DPP), made the phenotypic identification more precise and faster (Ubbens and Stavness., 2017) (Table 1).

**TABLE 1:** List of online software packages and algorithms for crop multi-omics analysis.

Software	Supported omics platform	Core algorithm	Availability/URL	Reference
CARMO	Genomics and transcriptomics	Database	http://bioinfo.sibs.ac.cn/carmo	Wang et al. (2015)
DCT	Integration of transcriptome data from different tissues	Joint correlation non-negative matrix factorization (jcNMF)	https://github.com/ztpub/DCT	Hu et al. (2020)
DPP (deep plant phenomics)	Phenomics	Neural networks		Ubbens and Stavness, (2017)
GpemDB	Genome, phenomics, transcriptome, proteome, metabolome, and enviromics	Scalable entity-relationship model		Gong et al. (2022)
IOMA	Proteomics and metabolomics	Quadratic programming (QP)		Yizhak et al. (2010)
KBCommons (knowledge base commons)	Phenomics, epigenomics, genomics, transcriptomics, proteomics, and metabolomics	Database	https://kbcommons.org	Zeng et al. (2019)
OmicsPLS	Metagenomics, transcriptomics, proteomics, and metabolomics	Two-way orthogonal PLS (O2PLS)	https://github.com/selbouhaddani/OmicsPLS	Bouhaddani et al. (2018)
Plant regulomics	Epigenomics, genomics, transcriptomics, and proteomics	Database	http://bioinfo.sibs.ac.cn/plant-regulomics	Ran et al. (2020)
PMN (plant metabolic network)	Database that integrates genomics, proteomics, and metabolomics	Database	https://plantcyc.org/	Hawkins et al. (2021)
MCIA	Transcriptomics, epigenomics, and proteomics data	Multiple co-inertia analysis	https://rdrr.io/bioc/omicade4/	Meng et al. (2014)
mixOmics	Metagenomics, transcriptomics, proteomics, and metabolomics	Partial least square-discriminant analysis (sPLS-DA)	http://mixomics.org/	Rohart et al. (2017)
MLLASSO	Transcriptome and metabolome	Multilayered least absolute shrinkage and selection operator		Hu et al. (2019)
MODAS	Genotypic data associated with mRNA transcripts and metabolic compounds	Dimensionality reduction (DR), Regional association (RA), Mendelian randomization (MR)		Liu et al. (2022)
SNF	Integration of DNA methylation, mRNA, and miRNA expression	Computes and fuses similarity networks	https://github.com/maxconway/SNFtool	Wang et al. (2014)
TOP (target-oriented prioritization)	Genomics and phenomics	Target-oriented prioritization with machine learning	https://github.com/yingjiexiao/TOP	Yang et al. (2022)

## Integration of genomics and transcriptomics

Generally, the candidate gene interval detected by GWAS or QTL mapping was large. Furthermore, combining with transcriptome data, analyzing the expression level of the candidate genes could better determine the key genes for complex quantitative traits. Using the integrative approach of GWAS for upland rice with transcriptomic profiles, the natural variation in the promoter of *DROUGHT1* (*DROT1*) was identified to confer drought resistance in rice (Sun et al., 2022). *MADS26* was identified by GWAS and transcriptomic to affect seed germination in maize (Ma et al., 2022). Moreover, integrating genetic and transcriptomic analysis in potato has fine mapped the *Ro* locus for tuber shape (Fan et al., 2022). Combining BSA-seq and RNA-seq, a *WOX* gene associated with plant architecture in rapeseed was identified for the compact phenotype (Ye et al., 2022).

GWAS combined with transcriptome-wide association study (TWAS) was also recently developed to detect causal genes for agronomic traits, which explained more heritable variation. Integrating GWAS and TWAS, the genetic basis of seed oil content and glucosinolate content were revealed in rapeseed (Tang et al., 2021; Tan et al., 2022). In maize, the genetic architecture of leaf cuticular conductance was elucidated by GWAS and TWAS (Lin et al., 2022). The expression QTL (eQTL) analysis is the association between the genetic variant and gene expression, which is also an important tool to elucidate complex phenotypes. Combining GWAS and TWAS with the eQTL analysis, the genetic regulatory network for cell wall biosynthesis in cotton has been uncovered (Li et al., 2020). Integrated QTL and eQTL mapping revealed candidate genes for fatty acid composition and flowering time in *Brassica napus* (Li et al., 2018a).

## Integration of genomics, transcriptomics, and metabolomics

The genome-wide association study based on metabolomics analysis (mGWAS) is another powerful complementary tool for phenotypic trait mapping, which has been widely used in crops, including rice, maize, wheat, barley, and tomato (Chen et al., 2014; Zhu et al., 2018; Chen et al., 2020; Zeng et al., 2020). The information of metabolites and metabolic pathways with genetic variations could elucidate the metabolic diversity and their relevance to complex traits for metabolomics-associated breeding in crops (Chen, et al., 2021).

Integrated metabolomics and transcriptomics analysis can establish the metabolite–gene correlation network and screen candidate genes for involving the metabolic pathways (Wen et al., 2014). For instance, the non-targeted metabolomics analysis of leaves for 385 maize lines was conducted with the eQTL analysis to identify drought tolerance hub genes (Zhang et al., 2021). Combined genomes, transcriptomes, and metabolomes reveal how the fruit metabolite content alterations occur in tomatoes during breeding. Also, the results suggested that the selection of genes associated with larger fruits changed the metabolite profiles, and the selection of five major loci that reduced antinutritional compounds rendered the fruit more edible (Zhu et al., 2018). Moreover, through RAD-seq and mGWAS *via* integrating metabolome and transcriptome data, novel candidate genes for seed coat color were identified, revealing the molecular mechanism of yellow seed in *Brassica rapa* (Zhao et al., 2022). Integrating QTL mapping, transcriptome, and metabolic profiling, two homologs of *EIN4* and *TRN1* in loquat were identified as promising candidate genes for fruit weight (Peng et al., 2022).

## Integration of genomics, transcriptomics, and microbiomics

The plant microbiome is the basis of plant growth and stress tolerance, including drought and disease resistance. The rhizosphere microorganisms which are the interface between root and soil, can not only promote the absorption of mineral nutrients by plants but also help plants resist the invasion of pathogens. The metagenomic analysis combined with transcriptome profiles revealed that soil microbiota affecting nitrogen metabolism contribute to the ultrahigh yield of rice (Zhong et al., 2020). Microbiome-wide association studies in cassava revealed *Lactococcus* sp. played an important role in disease resistance, which could be used for control of cassava disease (Zhang et al., 2021). Metagenomic and metatranscriptomic analyses of different watermelon cultivars suggested that the fruit-associated microbiome might play an important role in the carbohydrate metabolism of ripe fruits (Saminathan et al., 2018). Genome-resolved metagenomics with time-series root transcriptome implicated iron metabolism in the root microbiome dynamics in response to drought stress (Xu et al., 2021). Lately, using metagenomics information as an external quantitative phenotype with genomic and transcriptomic data, candidate genes in barley were identified for shaping microbiota composition (Escudero-Martinez et al., 2022). Also, in tomatoes, the bacterial genes involved in the metabolism of iron, sulfur, and vitamins were reported to associate with specific QTLs (Oyserman et al., 2022). The discovery of plant probiotics can increase crop yield, resist to biotic or abiotic stresses, and minimize chemical input (Levy et al., 2018). Thus, the microbiome could be another useful information to incorporate into crop breeding programs.

## Integration of genomics, epigenomics, and transcriptomics

Epigenetic variations are mainly dependent on the environments (e.g., biotic and abiotic stresses), which reprogram the transcriptome and maintain the genome stability to adapt to dynamic environments. Epigenetic diversity could produce new heritable phenotypes to widen the source of genetic and phenotypic variations, which has potential for crop improvement (Tirnaz and Batley, 2019; Hou and Wan, 2021). Integrating epigenomic information and transcriptome in 20 representative rice varieties provides comprehensive rice functional DNA elements maps for transcriptional regulation (Zhao et al., 2020). The epigenome maps combined with transcriptomes of *B. napus* also lay a foundation for the genetic regulation of traits in crop improvement (Zhang et al., 2021). Epigenetic modifications can regulate fertility transition and heterosis *via* altering the gene expression (Hu et al., 2015). Global DNA methylation, transcriptome, and small RNA profiling analysis revealed the regulatory networks and genes related to hybrid vigor in pigeon peas (Sinha et al., 2020). In wheat, through comprehensive analyses of the open chromatin, DNA methylome, and transcriptomic data elucidated the roles of *cis*-regulatory elements affecting transcription on a genome scale (Li et al., 2019). Also, combined with nascent RNA sequencing and epigenome profiling, the active transcription of enhancers in the wheat genome was revealed to regulate gene expression (Xie et al., 2022). Comparing the epigenomes and transcriptomes from various tissues under different developmental and environmental conditions provides valuable resources for regulomics in wheat (Wang et al., 2021). In addition, the epigenome and transcriptome changes in response to *Magnaporthe oryzae* infection implied that epigenomics is involved in rice fungal pathogens (Cui et al., 2021). Therefore, epigenetic variations can be used to reprogram their transcriptome for balancing the various important agronomic traits, and epigenetic diversity is a necessity in crop breeding programs (Tirnaz and Batley, 2019).

## Integration of other multi-omics approaches

To comprehensively reveal the potential mechanism at genetic and protein levels in crops under biotic and abiotic stresses, transcriptomics and proteomics were often used to analyze the global changes. Transcriptomics and proteomics analysis of soybean symbiosis with arbuscular mycorrhizal fungi (AMF) gives some insights into the molecular basis of disease resistance (Zhang et al., 2020). Using transcriptomics- and proteomics-based screening, small secreted proteins (SSPs) were identified to regulate rice immunity by rice blast (Wang et al., 2020). Integration of proteomic and transcriptomic profiles, systematic salt tolerance in cotton, and the alternative molecular network of N-deficiency stress in rice were revealed (Peng et al., 2018; Liang et al., 2021). In addition, the genome-scale metabolic pathways integrated with other databases (eg., PMN) were constructed (Hawkins et al., 2021) (Table 1).

Integrated gene regulatory network of microRNAs (miRNAs) and transcription factors (TFs) and genes revealed that intertwined miRNA-containing FFLs are associated with miRNA hubs in *Arabidopsis* (Gao et al., 2021). Lipidomic and transcriptomic analysis enable the understanding of citrus fruit quality maintenance (Wan et al., 2020). In rice, the genetic architecture of ionome variations has been elucidated *via* GWAS analysis of 17 mineral elements in grains (Yang et al., 2018).

## Integrative methodologies and databases

Due to a large amount of high-throughput data, multi-omics system biology, such as software tools, databases, and approaches are required for multi-omics integration. Recently, these large data sets can be comprehensively assimilated, annotated, as well as modeling using a systematic multi-omics integration (MOI) (Jamil et al., 2020). Machine learning (ML) and deep learning (DL) have been widely used to integrate omics datasets to reveal the functional relationships with these data. Lately, target-oriented prioritization (TOP) was developed to learn the inherent correlations among traits and balance the selection of multiple traits simultaneously (Yang et al., 2022) (Table 1). Several unsupervised clustering methodologies were developed to integrate the multi-omics data, such as GpemDB, IOMA, mixOmics, OmicsPLS, MODAS, multiple co-inertia analysis (MCIA), and similarity network fusion (SNF) (Yizhak et al., 2010; Meng et al., 2014; Wang et al., 2014; Rohart et al., 2017; Bouhaddani et al., 2018; Tini et al., 2019; Gong et al., 2022; Liu et al., 2022) (Table 1). This multiple molecular level (omics) data analysis can extract more knowledge from the available data. Thus, we should consider integrating the omics data simultaneously and reduce false-positive results by adding a priori information (Tini et al., 2019).

A comprehensive database integrates multi-omics data from the same crop and provides a valuable resource for gene cloning or study of the regulatory network, promoting crop improvement, such as MBKbase, WheatOmics, ZEAMP, and so on (Gui et al., 2020; Peng et al., 2020; Ma et al., 2021). On the other hand, many web database platforms were also developed to explore the functional information from multi-omics data, including CARMO, OmicsAnalyst, MapMan4, KBCommons, and Plant regulomics (Wang et al., 2015; Schwacke et al., 2019; Ran et al., 2020; Zeng et al., 2020; Zhou et al., 2021). Lately, an integrative platform in the ENCODE standards, ChIP-Hub, has provided rich resources from the regulome and epigenome data in plants (Fu et al., 2022). Moreover, the integration of regulome and genetic variations leads CRISPR-cereal to promote precise gene editing for wheat, rice, and maize (He et al., 2021). In addition, integrative pipelines for transcriptome or epitranscriptome sequencing data could also offer a clue to the discovery of candidate genes. For instance, to identify the key genes across multiple tissues for yield in rice, a novel dynamic cross-tissue (DCT) network analysis based on the transcriptome was developed to map genotype to phenotype by gene networks (or modules), namely, genotypes → network → phenotypes (Hu et al., 2020). Also, deepEA is a containerized web server for the integration of epitranscriptome with different chemical modifications, including 5-methylcytidine (m^5^C), N^6^-methyladenosine (m^6^A), and so on (Zhai et al., 2021). Using an algorithm, gene co-expression network differential edge-like transformation (GRN-DET), the key regulatory miRNAs for plant development and important traits can be identified by co-variances of miRNA-mRNA (Hu et al., 2018).

Since the features of the large scale, high dimension, high noise, and strong heterogeneity of multi-omics data, more software or algorithms should be developed for gene discovery in crop improvement.

## Discussion and future perspectives

**FIGURE 1:**
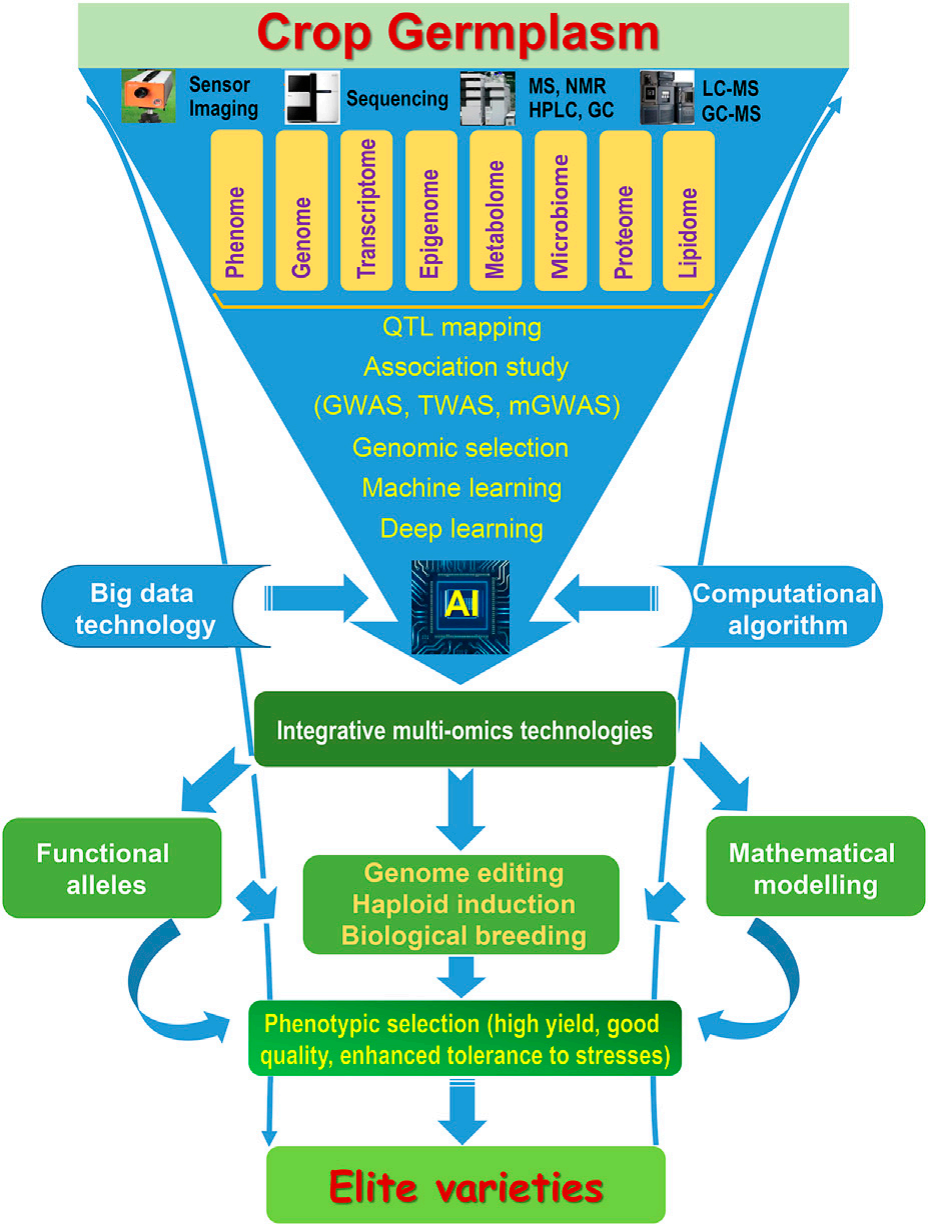
Integration of multi-omics technologies accelerates crop improvement. MS, mass spectrometry; NMR, nuclear magnetic resonance; HPLC, high-performance liquid chromatography; GC, gas chromatography; AI, artificial intelligence.

Due to NGS technologies generating large-scale sequence data, the collation and utilization of this vast data would require an interdisciplinary approach to integrate, which will be precise tools for crop improvement. GS with doubled-haploid (DH) technology (haploid breeding) could accelerate the breeding process to obtain the elite varieties (Hu et al., 2019; Fu et al., 2022). Multi-omics technologies including genomics, transcriptomics, proteomics, and metabolomics can link the genotype and phenotype, and integrate more information for systematic analysis to largely identified candidate genes for crop improvement (Figure 1). Multi-omics data will be analyzed in a more systematic and integrated way for accelerating crop improvement, such as an intelligent seed selection system. When integrating the multi-omics datasets, “phenotype to genotype” and “genotype to phenotype” as well as the genotype and environment interaction should be considered with the system biology approach to provide the basis for crop genetic improvement. Epigenetic diversity should also be considered in crop breeding programs due to desirable phenotypes by epigenetic modifications. *De novo* domestication based on the high-quality genome could speedily domesticate wild crops with retaining genetic diversity and elite alleles, which is a new breeding strategy to meet future agricultural challenges, such as rice and tomato (Li et al., 2018b; Yu et al., 2021). The utilization of omics technologies with genome editing, genomic selection, and haploid induction augmented by multi-scale “pan-omics” data will promote crop improvement to obtain a high yield, good quality and to enhance the tolerance of stresses (Figure 1) (Ghosh et al., 2018, Weckwerth et al., 2020).

There is a challenge to integrate disparate data from different platforms and formats across the genotype–phenotype spectrum as well as analyze and interpret the final results. The other problem is how to improve the phenotypic prediction based on the large-scale multi-omics data. Novel algorithms or models should also be developed to predict heterosis or complex phenotypes with AI including machine learning and deep learning (Dan et al., 2021; Wang et al., 2021). Thus, integration of more robust visualization tools, multi-omics analysis approaches, statistical genetic models, bioinformatics tools, and cloud computing with interdisciplinary should be integrated to explore candidate variations underlying agronomic traits.

In the future, a promising frontier is the integration of multi-omics data on the single cell level, single-cell multi-omics, which has great potential for crop improvement. The single-cell analysis will be used to identify non-anatomical markers for various cell populations and map individual cell stages during the differentiation of crop plants (Luo et al., 2020). With the development of single-cell multi-omics technologies, it will be possible to conduct simultaneous analyses of the genome, transcriptome, metabolome, and epigenome from a single cell (Shaw et al., 2021). These multi-omics integrative analyses of single cells provide valuable information on how genotype to phenotype at the single-cell level occur.

## Data Availability

The datasets presented in this article are not readily available. Requests to access the datasets should be directed to hujh05@nwafu.edu.cn.
